# Microstructural and Radioactive Shielding Analyses of Alumix-231 and Alumix-231 Reinforced with B_4_C/SiC/Al_2_O_3_ Particles Produced through Hot Pressing

**DOI:** 10.1021/acsomega.3c03132

**Published:** 2023-09-21

**Authors:** Uğur Gökmen, Leili Eslam Jamal Golzari, Seda Gürgen Avşar, Zübeyde Özkan, Sema Bilge Ocak

**Affiliations:** † Faculty of Technology, Department of Metallurgical and Materials Engineering, Gazi University, Ankara 06500, Turkey; ‡ Graduate School of Natural and Applied Sciences, Department of Advanced Technologies, Gazi University, Ankara 06500, Turkey

## Abstract

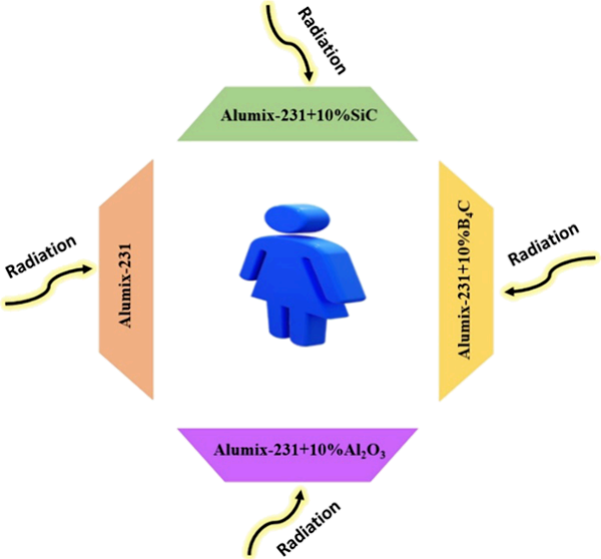

Al_2_O_3_, SiC, and B_4_C (10%) particle-reinforced Alumix-231 matrix composites and nonreinforced Alumix-231 blocks were produced by pressing under uniaxial pressure using the powder metallurgy method. The Archimedes density of the produced samples was analyzed using microstructures (SEM and EDS), powder size analysis, and theoretical (PSD software) and experimental methods (Co-60 and Cs-137 radiation sources). As a result of the theoretical and experimental calculations, the Alumix-231 + 10% B_4_C composite material showed the lowest shielding feature against γ radiation, while the Alumix-231 + 10% Al_2_O_3_ composite material showed the highest shielding feature.

## 1 Introduction

Radiation is the emission of energy in the form of waves or particles. Radiation can be natural or man-made. Radiation can be classified into several types based on their origin, properties, and effects. We can list the common types of radiation: electromagnetic radiation (X-rays), ionizing radiation (γ rays), nonionizing radiation (radio waves), particulate radiation (alpha particles), natural radiation (the sun), and artificial radiation (nuclear power plants).^1−4^ The classification of radiation is important for understanding its properties and potential effects on living organisms and the environment. Different types of radiation require different shielding and safety precautions. This happens since some forms of radiation can be harmful to living organisms, particularly if they are exposed to high levels over an extended period of time. Radiation shielding refers to the use of materials to protect people and objects from harmful effects of radiation. The shielding material absorbs or deflects the radiation, reducing its intensity and protecting living organisms and military equipment.^5^ The effectiveness of radiation shielding depends on factors, such as the type and energy of the radiation, the thickness and density of the shielding material, and the distance between the radiation source and the object being shielded. Radiation shielding is important in a variety of applications, such as nuclear power plants and space exploration.^6,7^ It is designed to protect workers, soldiers, patients, and the general public from the harmful effects of radiation exposure while still allowing for the safe use of radiation in various applications. For instance, different levels of certain types of radiation, for example, used in medical imaging, can be beneficial for the diagnosis and treatment of illnesses.^6,7^ Prevalent materials used for radiation shielding include lead, boron, concrete, bismuth, stainless steel, and water.^6−10^ However, nowadays, composite materials have started to attract attention for radiation shielding studies^11^ since composite materials offer lightness thanks to their lower density while providing higher mechanical properties than concrete.^12^ In addition, the low melting temperature, production of poisonous substances, and lower protection capacity against high energy radiation highlight the use of composite materials instead of lead.^11^ Metal matrix composites (MMCs) are materials consisting of a metal matrix reinforced with one or more secondary phases such as ceramic particles, fibers, or whiskers. The preparation methods for MMCs can vary depending on the desired composite structure and properties. Some common techniques used to prepare metal matrix composites are liquid metal infiltration, stir casting, in situ reinforcement, and solid-state processing, and these are traditional fabrication processes. In processes like casting, the presence of nanoscale reinforcements can lead to uncontrolled and undesired clumping of particles into larger clusters. These clusters are unevenly distributed within the matrices due to strong van der Waals forces among the nanosized particles.^13−15^ According to Cai et al., selective laser melting (SLM) is considered as a new and promising technology for metal matrix composites including nanoscale reinforcement.^13,14^

Another common but advantageous method for our study, the MMC production method, is powder metallurgy (PM). Powder metallurgy is a promising and efficient technique that offers energy efficiency and economic viability for manufacturing simple as well as intricate components with precise dimensions.^16^ Our team preferred to benefit from the advantages of powder metallurgy as it worked with macrosized powders in the experiments conducted in this study. This method involves the blending of metal powders with the desired reinforcement materials. The mixture is then compacted into a preform followed by a sintering process to bond the particles together. Additional heat treatments may be applied to enhance the matrix-reinforcement interface and improve the composite properties.^16^ It is important to note that these are general methods for preparing metal matrix composites, and specific variations and modifications may exist depending on the desired composite composition, geometry, and application. The selection of the appropriate preparation method depends on factors, such as the desired properties, cost considerations, and the nature of the reinforcement and matrix materials being used.

Moreover, the biggest advantage of composite materials is design. Various composites of the same material can be used, which have varying operating conditions, according to different industrial areas. The change in the physical and mechanical properties of the same material allows the desired design to be made by using matrix and reinforcement materials of different compositions. Among the metal materials used, aluminum metal has a very important place due to its easy manufacturability and low temperatures.^17−21^ Additive to its capabilities, aluminum metal also has significant disadvantages such as low melting temperature and mechanical strength.^17−21^ To eliminate the disadvantages of aluminum and to make it a material with higher mechanical properties, different aluminum alloy series are produced by alloying with other elements.^17−21^ One of the produced alloy series is the Alumix series.^17−21^ The main element of the Alumix series is aluminum. It has different names according to the alloying elements that it contains. The most commonly used aluminum alloys in the Alumix series are Alumix-123 (Al, Cu, Mg, and Si), Alumix-321 (Al, Mg, and Cu), Alumix-231 (Al, Si, Mg, and Cu), and Alumix-431 (Al, Zn, Mg, and Cu).^17−21^ Due to possessing higher corrosion resistance and electrical conductivity and lower density, aluminum alloys are frequently used in different industrial areas.^22^ Furthermore, the enhancement of the mechanical properties of composite materials relies on the interaction between the reinforcement material and the matrix as well as the even distribution of the reinforcement phase within the matrix phase. Ceramic reinforcements, including SiC, B_4_C, TiC, and Al_2_O_3_, are commonly utilized in various applications.^23−25^ In addition to being used as a reinforcement element in metal matrix composite materials, ceramic materials are also used by some scientists as radiation shielding materials due to their high density and ability to absorb radiation. Aluminum oxide, also known as alumina, is often used in radiation shielding for its high density and ability to absorb γ rays.^26^ Titanium diboride is known for its high melting point, hardness, and ability to absorb neutrons. It is often used in nuclear reactors and other high-temperature applications.^27,28^ Boron carbide is commonly used in radiation shielding due to its high melting point, hardness, and ability to absorb neutrons.^8,29^ These ceramic powders can be used alone or combined with other materials to create effective radiation shielding. The specific choice of ceramic powder will depend on the type of radiation being shielded and the specific application.^30^

Alumix-231 (Al–Cu 2.5%–Mg 0.5%–Si 14%) commercial powder was used in the study. It is aimed to produce samples with higher strength and corrosion resistance due to the high Si ratio. SiC, B_4_C, and Al_2_O_3_ ceramic materials, which are commonly used to increase strength in composite materials, were used together in this study, and the usability of radiation shielding properties in composite materials was tried to be analyzed simultaneously.

This study is to investigate the gamma-ray attenuation behaviors of shielding materials of Alumix-231 and composite samples by using the Phy-X/PSD platform and radiation transmittance tests. In this research, the linear and mass attenuation coefficient (LAC and MAC), tenth-value and half-value layer (TVL and HVL), and mean free path (MFP) values of the B_4_C, Al_2_O_3_, and SiC (10 wt %) particle-reinforced Alumix-231 composites and Alumix-231 were theoretically and experimental calculated for the radiation shielding. Consecutively, all the values were calculated for these composite materials by running the Phy-X platform and comparing them with each other. In radiation permeability tests, Co-60 (1173 keV) and Cs-137 (662 keV) radioisotopes with medium energies, which are widely used in medicine and industry, were used as γ radiation sources. In addition to the theoretical and experimental radiation studies, the dimensional analyses of the powders used and the densities of the produced samples were calculated and compared using the Archimedes principle. Ultimately, the microstructures were visualized by scanning electron microscopy (SEM) and energy-dispersive spectrometry (EDS).

## 2 Composite Materials Production and Analysis

### 2.1 Composite Materials Production

**Figure 1:**
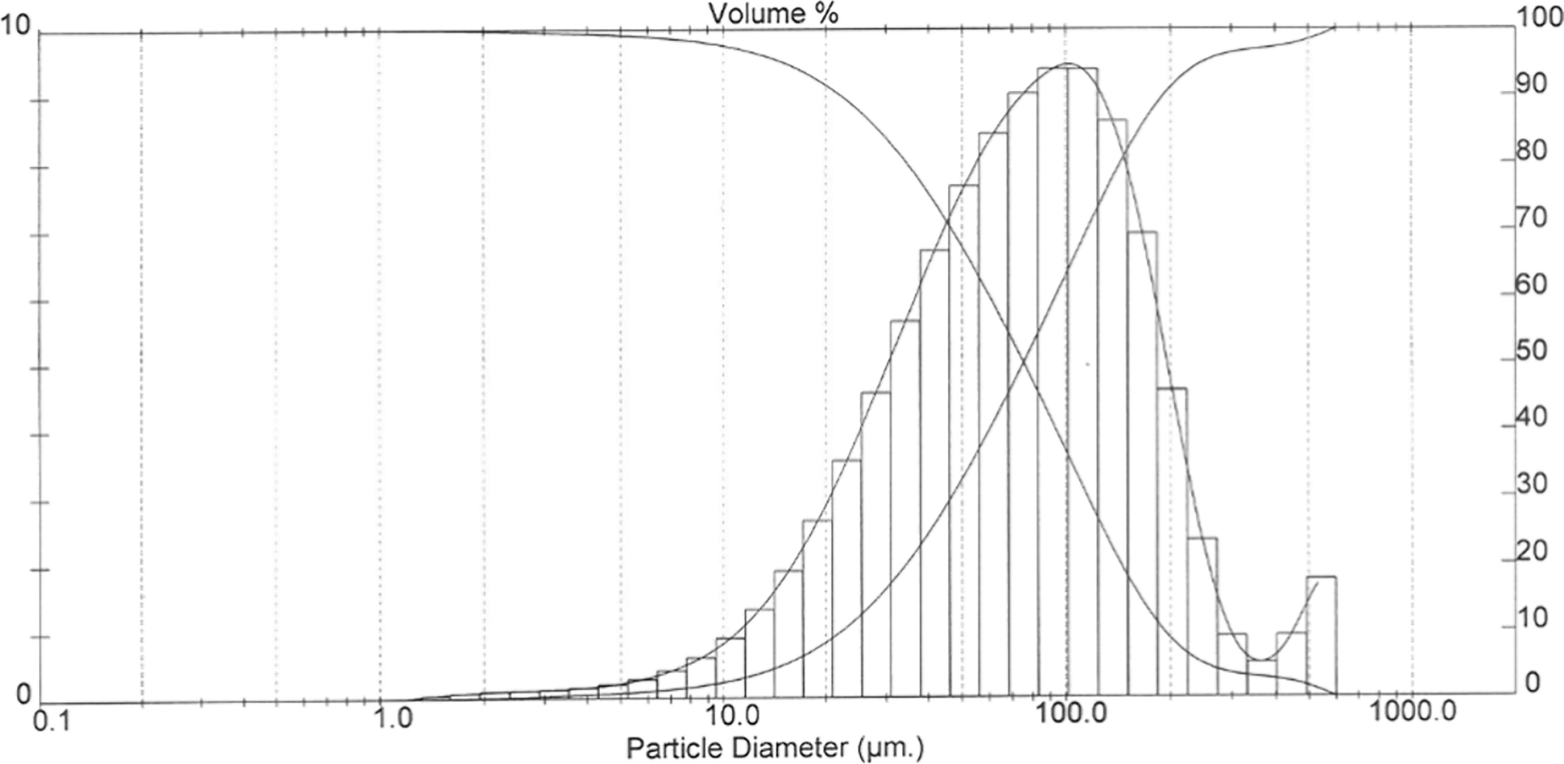
Alumix-231 powder size distribution.

**Figure 2:**
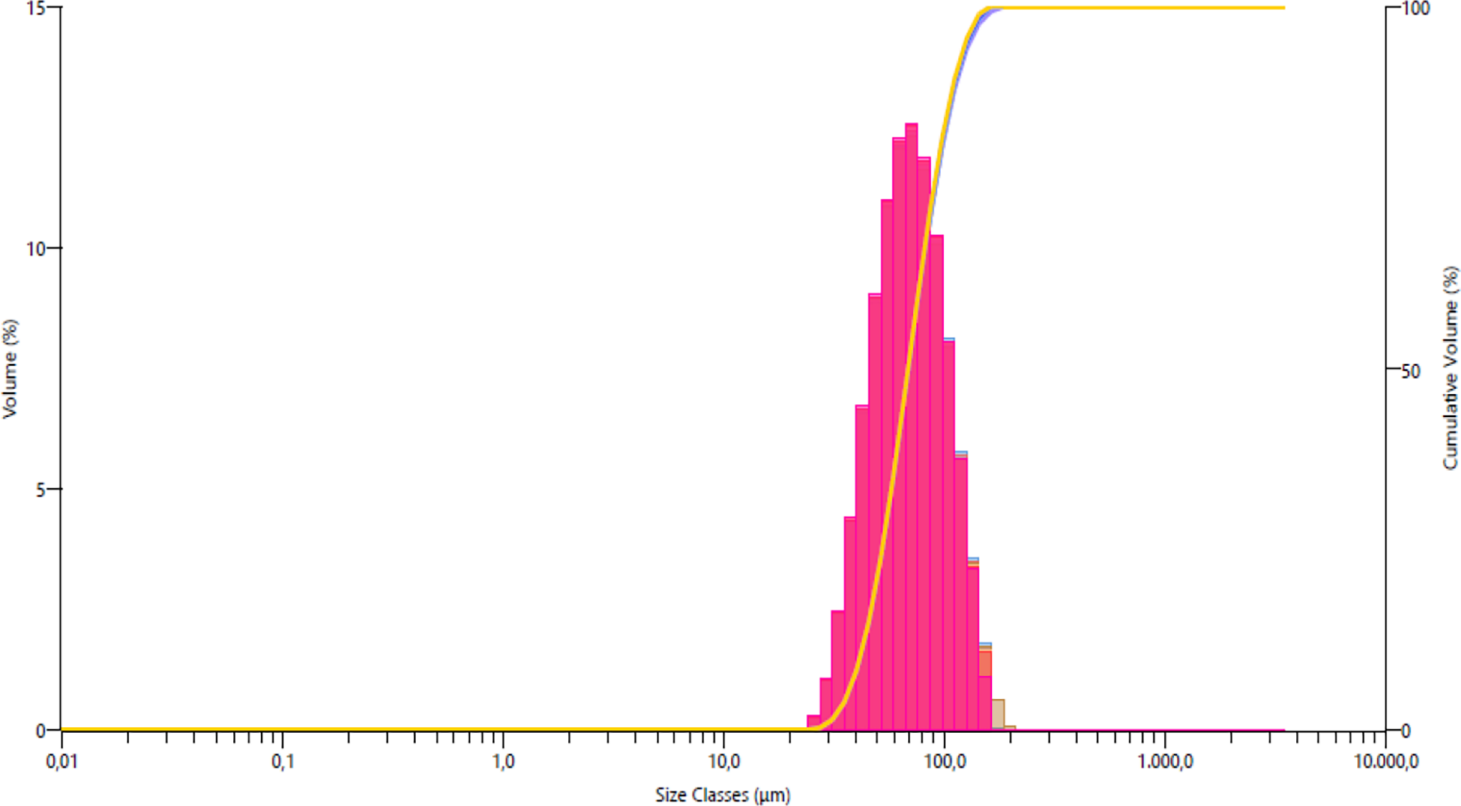
B_4_C powder size distribution.

**Figure 3:**
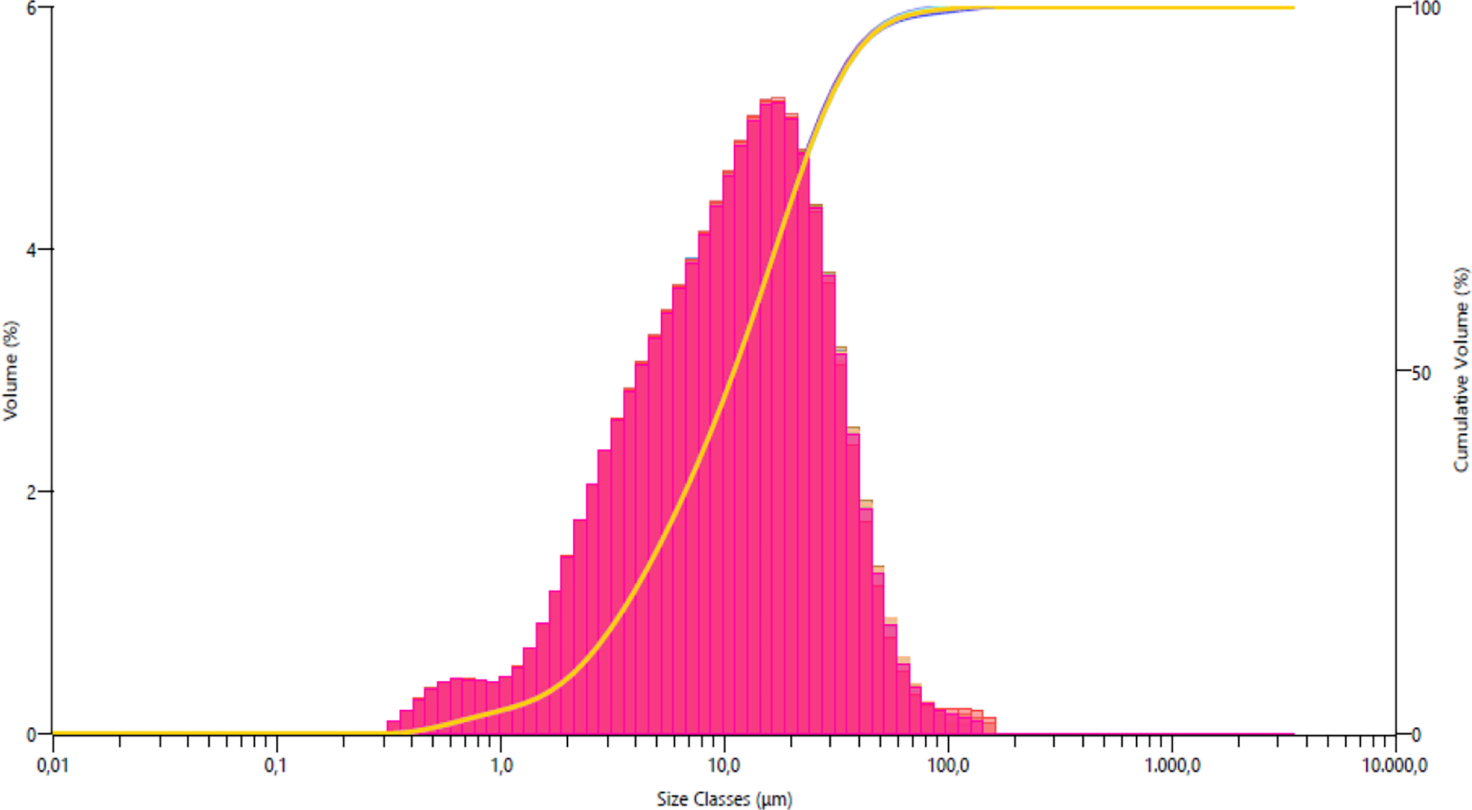
SiC powder size distribution.

**Figure 4:**
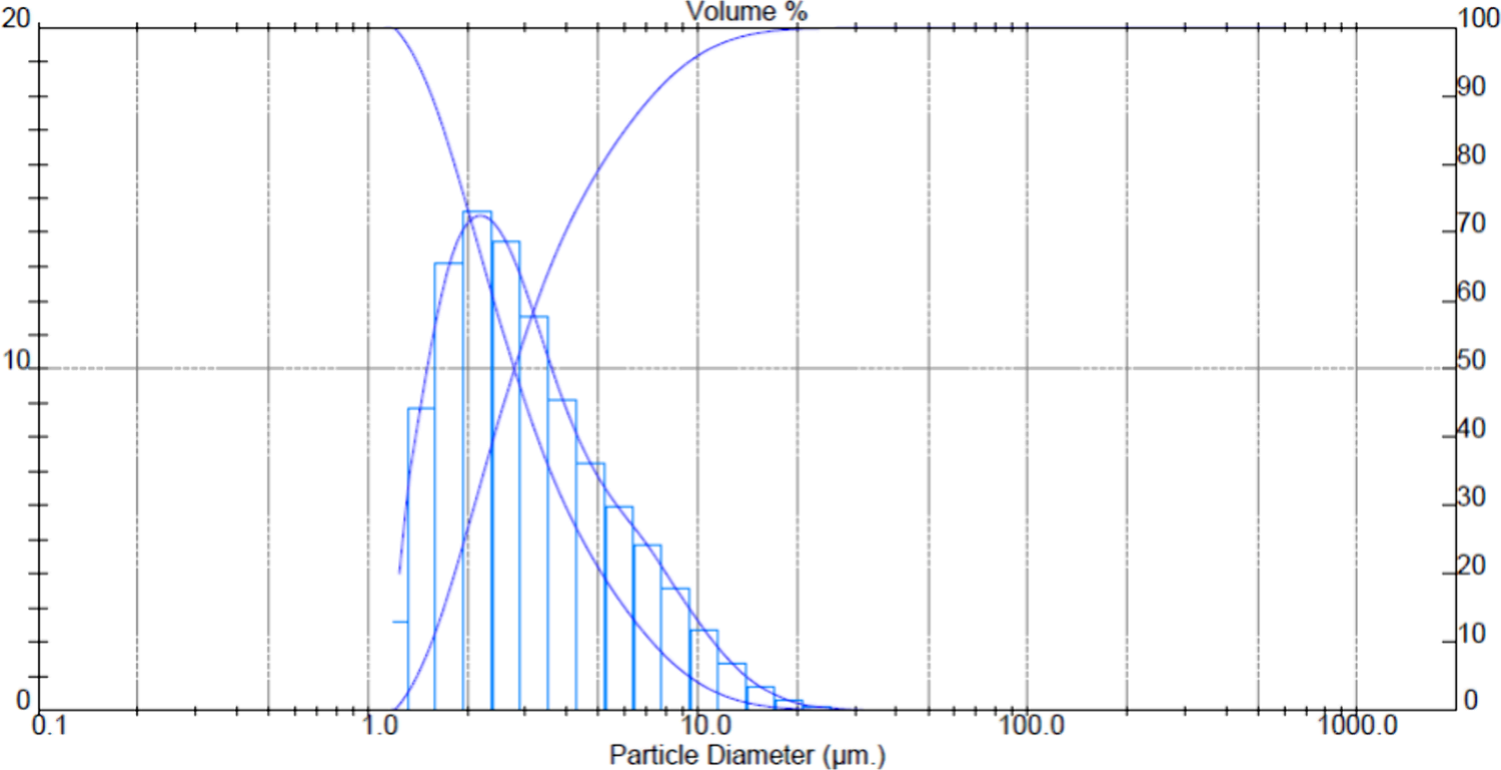
Al_2_O_3_ powder size distribution.

**Figure 5:**
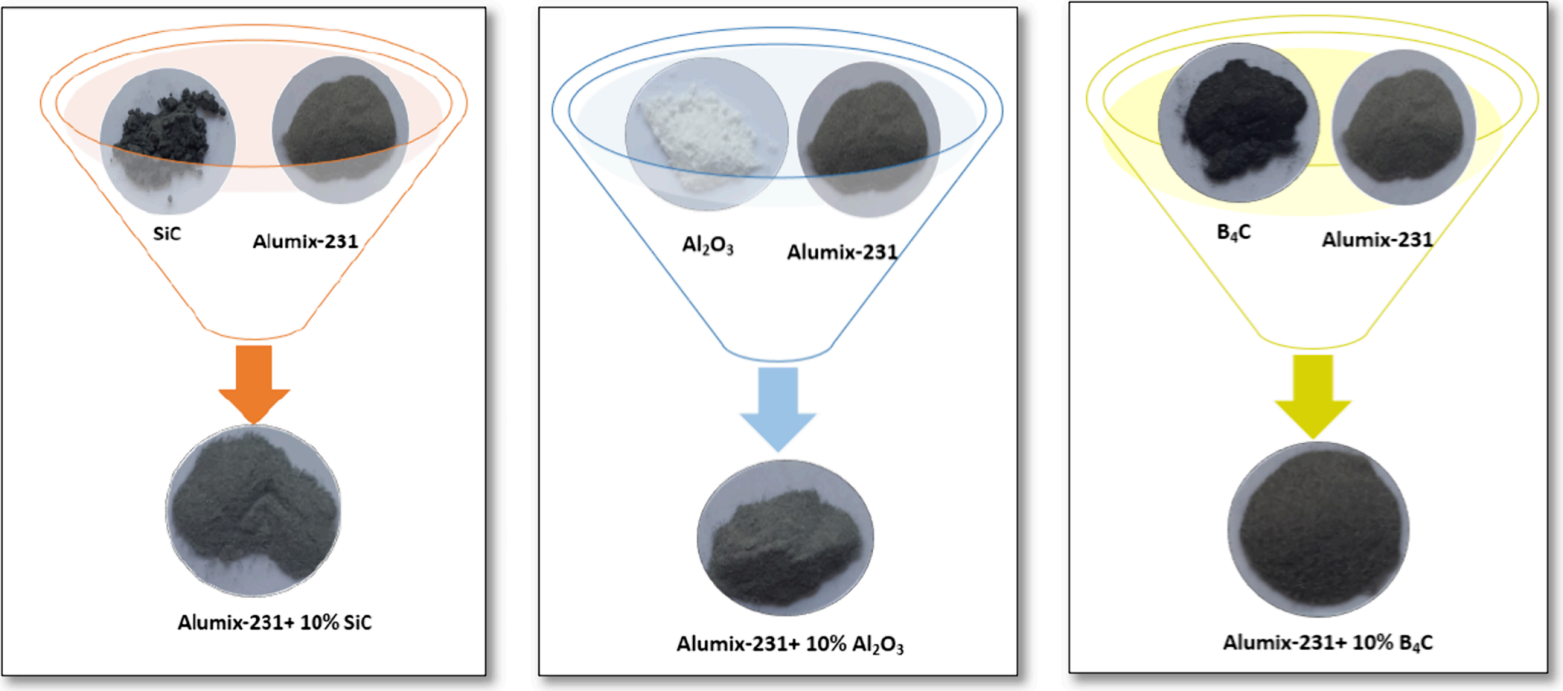
Mixture images of matrix and ceramic materials.

**Figure 6:**
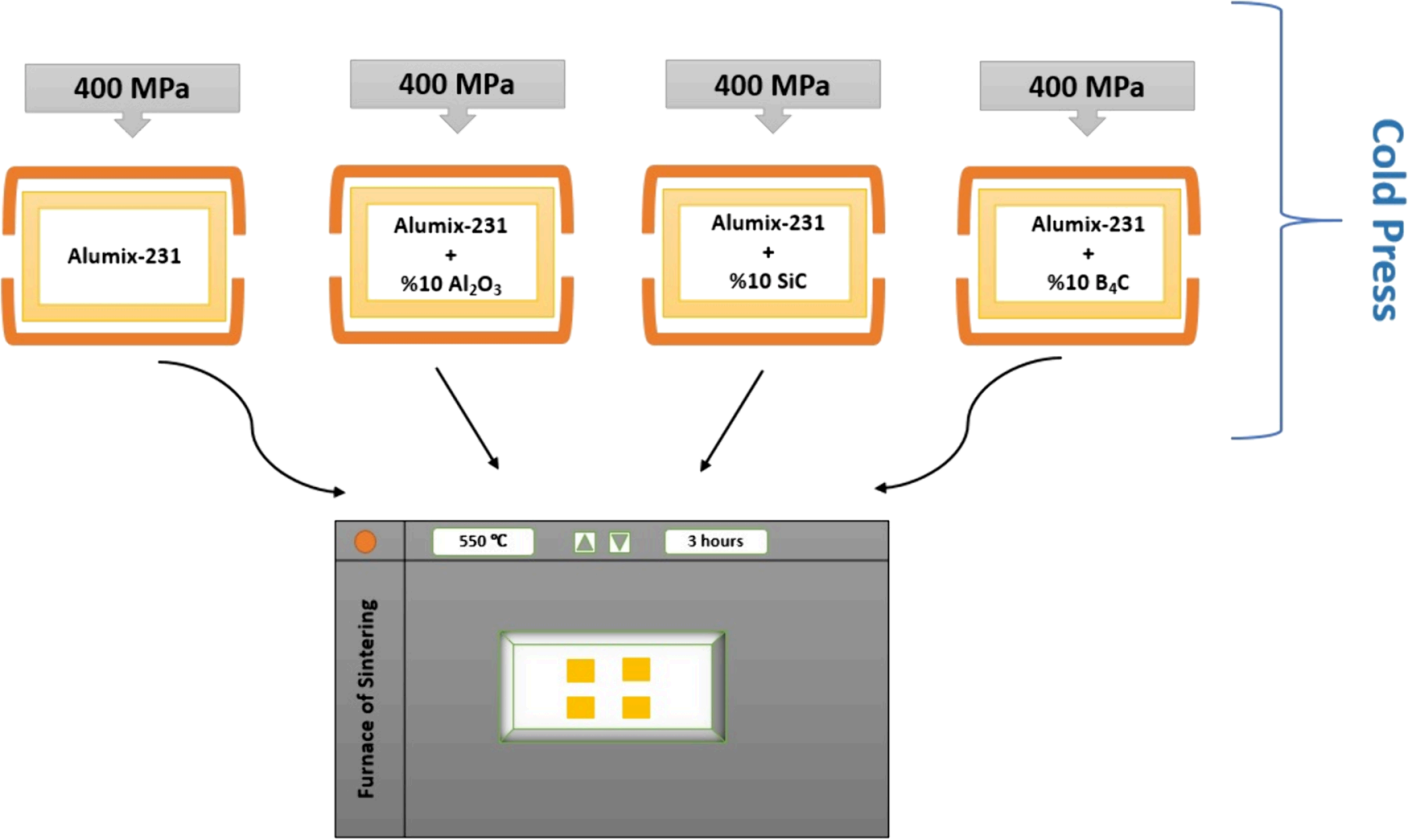
Production scheme of composite materials.

Alumix-231 used in this study was a hypereutectic prealloyed Al–Si P/M alloy powder material with a chemical composition of Al–Cu 2.5–Mg 0.5–Si 14 by % and the trade name of Ecka Alumix-231. The Alumix-231 matrix powder was obtained from Ecka Granules (Germany), and Al_2_O_3_, SiC, and B_4_C ceramic powders were obtained from Sigma-Aldrich (ABD). Powder size analyses of ceramic powders (B_4_C, SiC, and Al_2_O_3_) and Alumix-231 powders supplied at different times were performed in a Malvern Mastersizer device. The graphics of the dimensions of the powders used are given in Figures1–4. The *D* (50) values of Alumix-231, B_4_C, SiC, and Al_2_O_3_ powders are 75.48, 69.4, 11, and 2.78 μm, respectively. In this study, three different composite materials were produced (Figure 6). To obtain the composite materials, in the first step, the required powder amounts were calculated. After that, 10% Al_2_O_3_, 10% SiC, and 10% B_4_C ceramic powders by weight were added into Alumix-231 powder, which is the matrix material for each of the composite materials, and three mixtures were obtained. These were mixed in a three-dimensional mixer (TURBULA shaker mixer) for 30 min in order to provide a homogeneous distribution. The images after the mixing of ceramic (B_4_C, SiC, and Al_2_O_3_) powders mixed with the 10% Alumix-231 matrix material in the Turbola device are given in Figure 5. The cold pressing process was carried out under a pressure of 400 MPa using a one-way hydraulic press in the mold, and composite materials were produced. Then samples were placed in the PROTERM brand MUFLE type furnace and were subjected to sintering process at 550 °C for 3 h (Figure 6). The sintering temperature and time were adjusted according to the matrix material Alumix-231. In this context, the heating times were preferred according to the values below the melting temperature of the matrix material. The cooling of the composite materials was carried out at room temperature. Afterward, the composite materials produced were removed from the furnace, and analyses were carried out.

### 2.2 Experimental and Theoretical Studies

#### 2.2.1 Archimedes Density

The densities of 3 different composite materials and Alumix-231 blocks produced were calculated according to the Archimedes principle^31^ using a Sartorius brand balance with 0.1 mg precision. The formulas used in the calculations are given in eq 1.
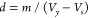
1

According to eq 1, *d* is the density (g/cm^3^), *m* is the weight (g), *V*_*y*_ is the wet weight (g), and *V_s_* expresses the weight (g) values in water.

#### 2.2.2 Phy-X/PSD

The Phy-X/PSD program was used to theoretically determine the γ radiation transmittance values of the materials, linear–mass attenuation coefficient (LAC–MAC), tenth–half-value thickness (TVL–HVL), etc., of the material. It is an online program that can theoretically analyze parameters related to radiation permeability in the energy range of 0.015–15 MeV.^32^

#### 2.2.3 Radioactive Permeability

GM-Counter/Geiger-Müller-Zahler PHYWE was used as a detector, and Cs-137 was used as a gamma source. Cs-137 has an activity of 1 μCi and emits 0.662 MeV of γ radiation. Likewise, Co-60 emits an activity of 1 μCi and a γ radiation of 1,332 MeV. As a detector, an HPGe detector (ORTEC/GEM50P4-83) was used with an analog-to-digital converter. Count times were taken as 5 min for the gamma sources used. For composite material measurements, measurements were taken by keeping the distance between the detector and the source constant at 7 cm.

The following parameters were examined in theoretical and experimental radiation permeability analyses.

##### 2.2.3.1 LAC–MAC

Linear and mass attenuation coefficients play a major role in the field of shielding and radiation protection. The linear attenuation coefficient (μ) is a constant describing the fraction of attenuated photons in a beam of energy per unit thickness of material. It shows the velocity of penetration of a beam of energy into the material. The value of the LAC relies on the energy of gamma photons, atomic number, and the density of shielding material. The eq 2 used to calculate LAC is as follows:
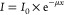
2where *I* denotes the intensity of radiation passing through the material, *I*_0_ is the intensity of radiation on the material, μ (cm^–1^) is the linear attenuation coefficient of the matter, and *x* (cm) is the thickness of material.^33,34^ The mass attenuation coefficient is the measurement of the interaction that might happen between the photons and matter. It is equivalent to the division of the linear attenuation coefficient (μ) by the density of the absorber (ρ), which is expressed in cm^2^/g.^32^ Since the mass attenuation coefficient is independent from density, it can be considered useful.^35,36^

##### 2.2.3.2 HVL–TVL

The half-value layer is the thickness of the material, which can reduce the intensity of radiation entering it to its half-value. The tenth-value layer is the thickness of the material, which can reduce the photon intensity to one-tenth of its original value by scattering or absorption.^37−39^ The half-value layer and tenth-value layer are calculated using eqs 3 and 4:
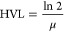
3
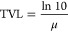
4

##### 2.2.3.3 MFP

The mean free path is a shielding characteristic that refers to the interaction of radiation with the atoms’ shielding material. It indicates the distance that the photons travel between two successive collisions.^40,41^ The MFP can be calculated using eq 5:
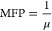
5

## 3 Results

Density values, SEM, EDS, Phy-x/PSD results, and experimental radiation results of SiC/B_4_C/Al_2_O_3_ particle-reinforced composite samples with the Alumix-231 matrix (at a 10% reinforcement ratio) and Alumix-231 samples without reinforcement material obtained as a result of theoretical and experimental studies were examined.

### 3.1 SEM and EDS

**Figure 7:**
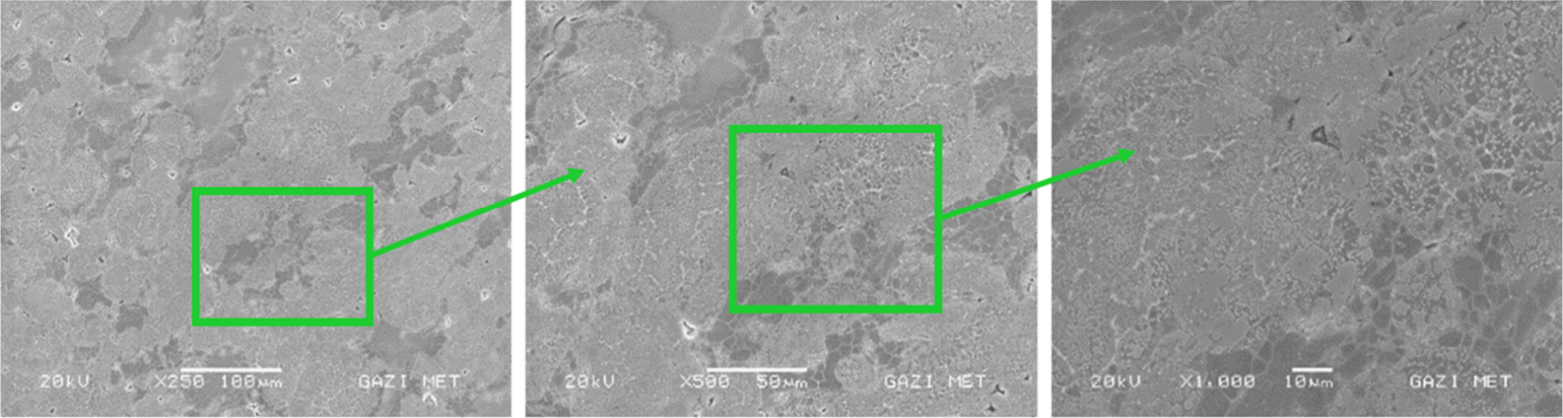
SEM images of Alumix-231 at 250×, 500×, and 1000×.

**Figure 8:**
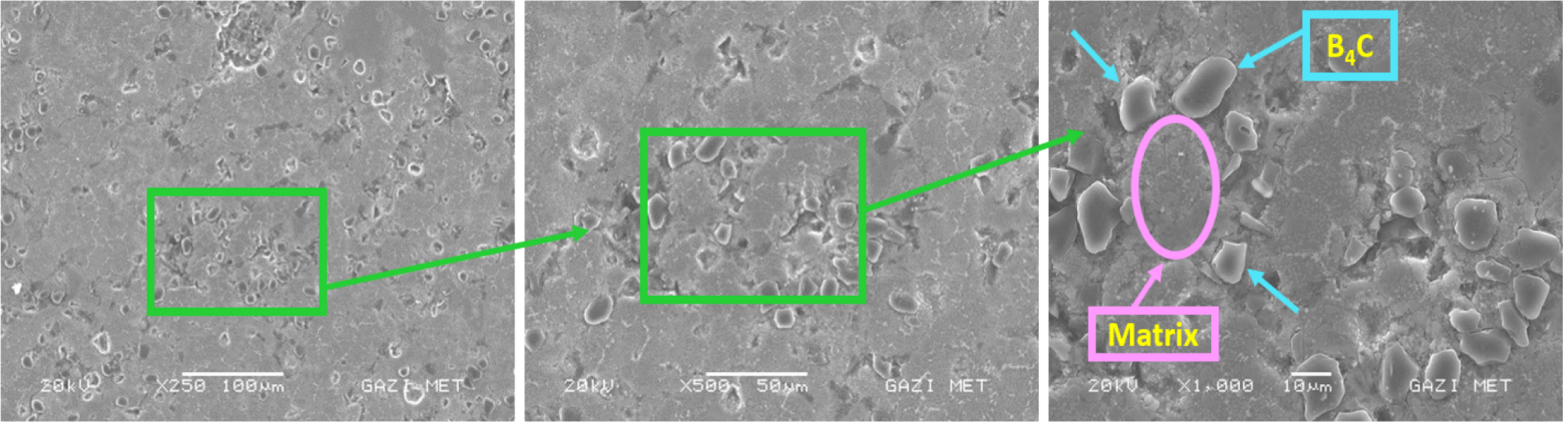
SEM images of Alumix-231 + 10% B_4_C at 250×, 500×, and 1000×.

**Figure 9:**
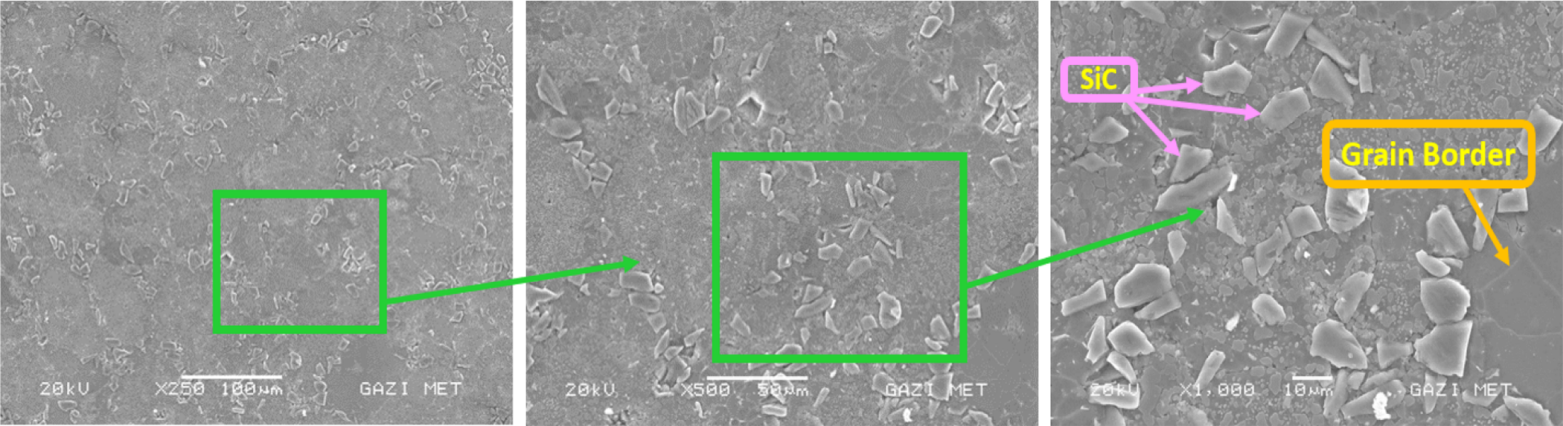
SEM images of Alumix-231 + 10% SiC at 250×, 500×, and 1000×.

**Figure 10:**
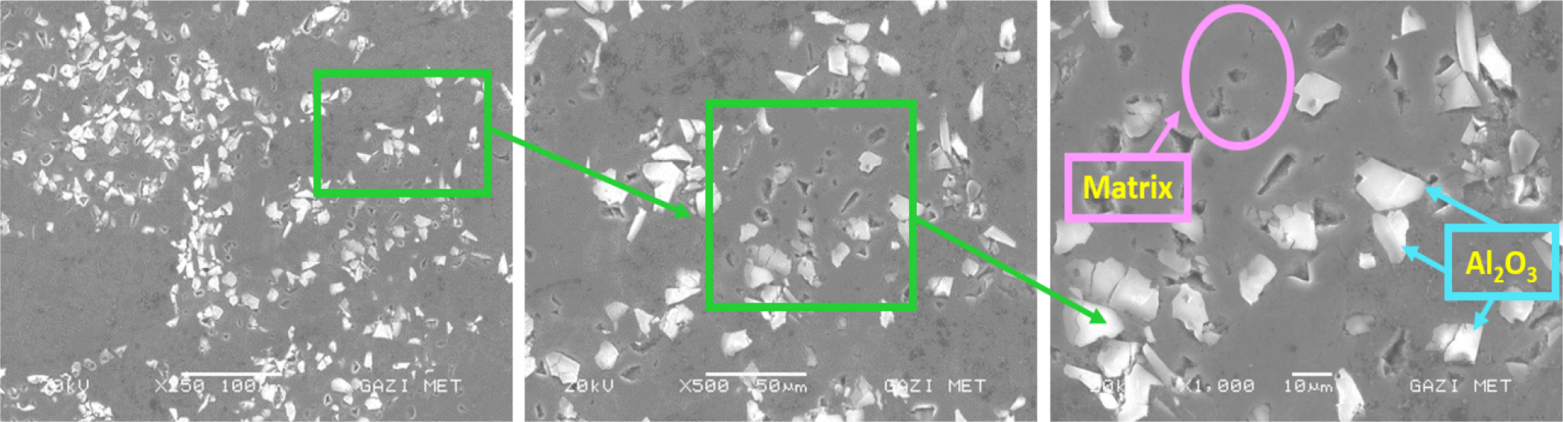
SEM images of Alumix-231 + 10% Al_2_O_3_ at 250×, 500×, and 1000×.

**Figure 11:**
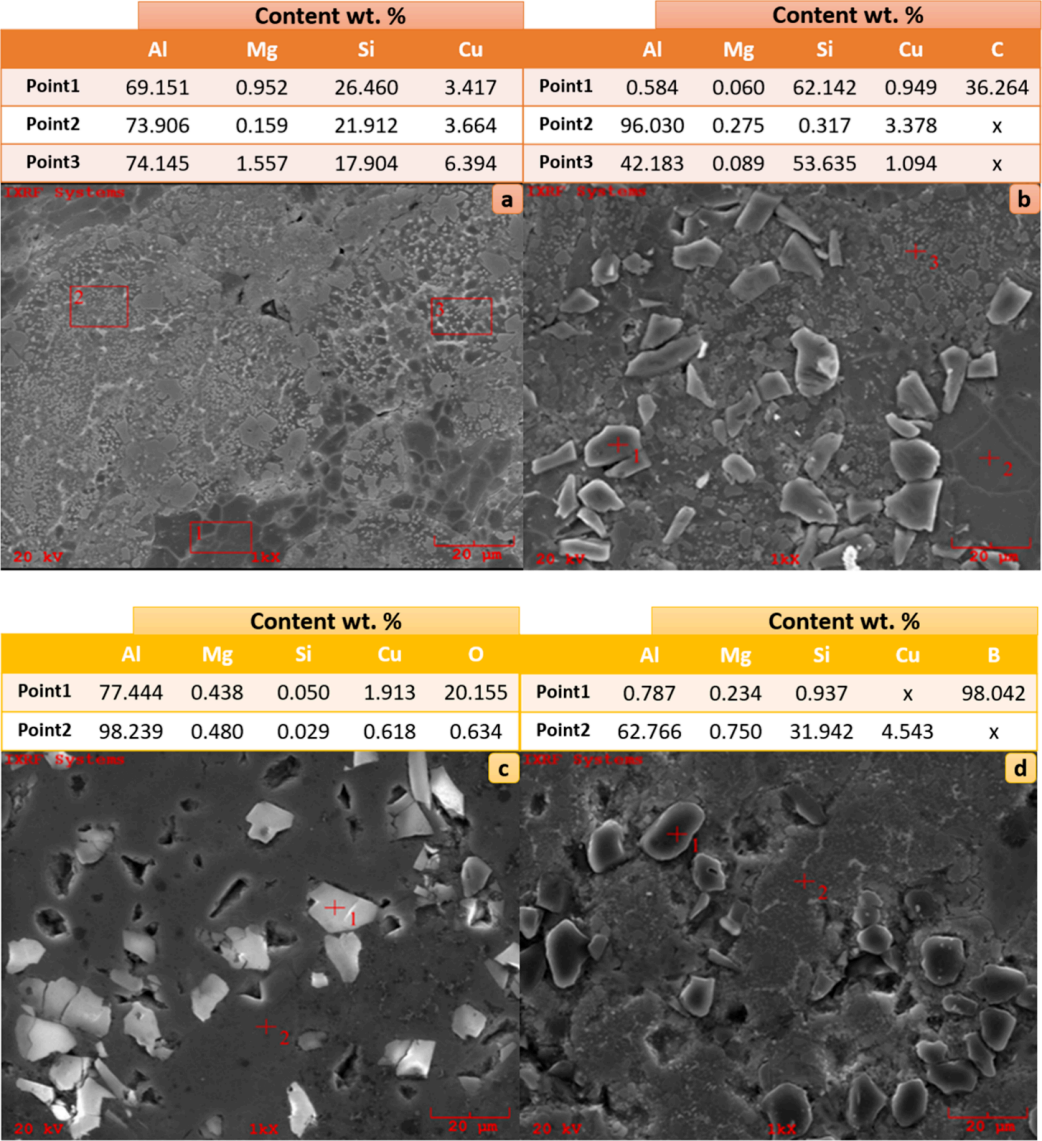
EDS images of (a) Alumix-231, (b) Alumix-231 + 10% SiC, (c) Alumix-231 + 10% Al_2_O_3_, and (d) Alumix-231 + 10% B_4_C.

The main alloying elements of the Alumix-231 alloy are Si–Cu–Mg. In Figures 7–10, in the SEM images of the Alumix-231 sample and composite materials produced by reinforcing 10% Al_2_O_3_, 10% B_4_C, and 10% SiC into the Alumix-231 matrix at 250×, 500×, and 1000× magnifications, it is seen that the powder grains approach each other and the pores are closed to a large extent. Al_2_O_3_ particles in the sample are lighter and brighter in color. Al_2_O_3_ particles were generally homogeneously distributed in the Alumix-231 matrix, although there were local agglomerations. Partial voids were observed at the interfaces formed between the Alumix-231 matrix and the B_4_C particles. It was observed that SiC (Figure 9) particles had more agglomeration in the Alumix-231 matrix compared with other composite samples. In Figure 8, it is seen that it is surrounded by the B_4_C reinforcement element added to the Alumix-231.When the EDS analyses taken at three points of the Alumix-231 nonreinforced material given in Figure 11a are examined, a high amount of Al is seen as the main element in each region where EDS is taken, as expected. It is clearly seen that the main alloying element in Alumix-231 is Si. When the EDS analyses taken from the Alumix-231 + 10% SiC composite given in Figure 11b are examined at three points, it can be understood from the Si and C ratios of the EDS at one point that it is a SiC particle. It is understood that it is Alumix-231 matrix material due to its high Al ratio at two points and matrix and reinforcement material together due to the high Al and Si ratio at three points. When the EDS analyses taken from the Alumix-231 + 10% Al_2_O_3_ composite given in Figure 11c are examined at two points, the oxygen ratio is high at one point where the EDS is taken, and as it can be understood from the particle shape, the powder particle at one point is the Al_2_O_3_ reinforcement material. When the EDS analyses taken from the Alumix-231 + 10% B_4_C composite given in Figure 11d are examined at two points, it is understood that the one point is B_4_C due to the high ratio of B, although the particle shape is not exactly clear due to the disappearance of the grain boundaries at the one point from which the EDS was taken.

### 3.2 Density

**Figure 12:**
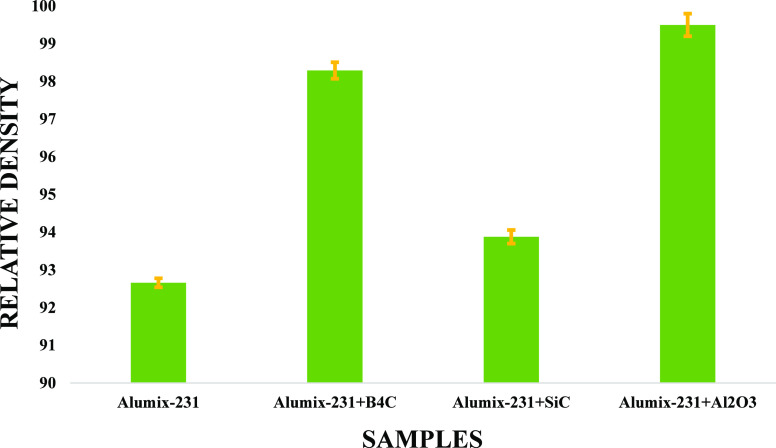
Alumix-231 samples and 10% Al_2_O_3_, 10% B_4_C, and 10% SiC reinforced composites with the Alumix-231 matrix.

It is possible to obtain different results depending on the properties of the materials produced by the powder metallurgy method, the processing conditions, and the powders used. In powder metallurgy, the density value of the material changes as the porosity rate of the produced materials changes during the sintering process. Theoretical density values and postsintering density values of the samples produced in Figure 12 were calculated using the Archimedean principle. Theoretical density values were higher than the density values obtained using the Archimedes principle.^42^ In powder metallurgy, the density of a sintered part plays a crucial role in determining its mechanical, physical, and radiation properties. The expected density in powder metallurgy refers to the theoretical maximum density that can be achieved during the sintering process. It is primarily influenced by the density of the individual powder particles and their packing characteristics. Theoretical models, such as the Hall–Petch equation, can provide estimates of the expected density based on particle size and shape parameters. The measured density, on the other hand, refers to the actual density obtained through experimental measurements of the sintered part. This can be determined using techniques like the Archimedes principle or by comparing the weight and volume of the part. The measured density may deviate from the expected density due to various factors, including porosity. Porosity is a critical parameter that affects the density of the sintered parts. Porosity refers to the presence of voids or empty spaces between the powder particles after sintering. Higher porosity leads to lower density because the voids occupy space that would otherwise be occupied by solid material. The correlation between density and porosity is generally inverse, meaning that as porosity increases, density decreases.^43,44^ It disregards the presence of crystal lattice defects, assuming that materials are perfectly crystalline at the theoretical densities. However, considering the fact that this is not the case, the data were higher than the data obtained as a result of experimental studies.

Among the samples produced, the lowest density material was Alumix-231 + 10%B_4_C with a value of 2.651 g/cm^3^, while the highest value was that of the Alumix-231 + 10%Al_2_O_3_ composite material with a value of 2.8 g/cm^3^. The sample with the lowest relative density among the samples produced was Alumix-231. The results of these measurements revealed how the packaging properties and molecular weights of the reinforcement elements affect the relative density. The results of these measurements revealed how the packaging properties and molecular weights of the reinforcement elements affect the relative density. Although the percentage values of the reinforcement elements in the composite material are equal, the highest value was found in alumina, while the lowest value was obtained in boron carbide. When the regions where the SEM images were taken are examined, it can be argued that the amount of pores in the Al_2_O_3_ reinforced structure is relatively higher than that in the carbide-containing composites. However, although the pores between the grains seem to be excessive, the detail that the SEM image is taken from a certain region of the part should not be ignored. The correlation between porosity and density is generally inverse, meaning that as porosity increases, density will decrease. In this context, we can suggest that the produced Al_2_O_3_ composite material has the smallest number of pores throughout the part.

### 3.3 Phy-x/PSD

**Figure 13:**
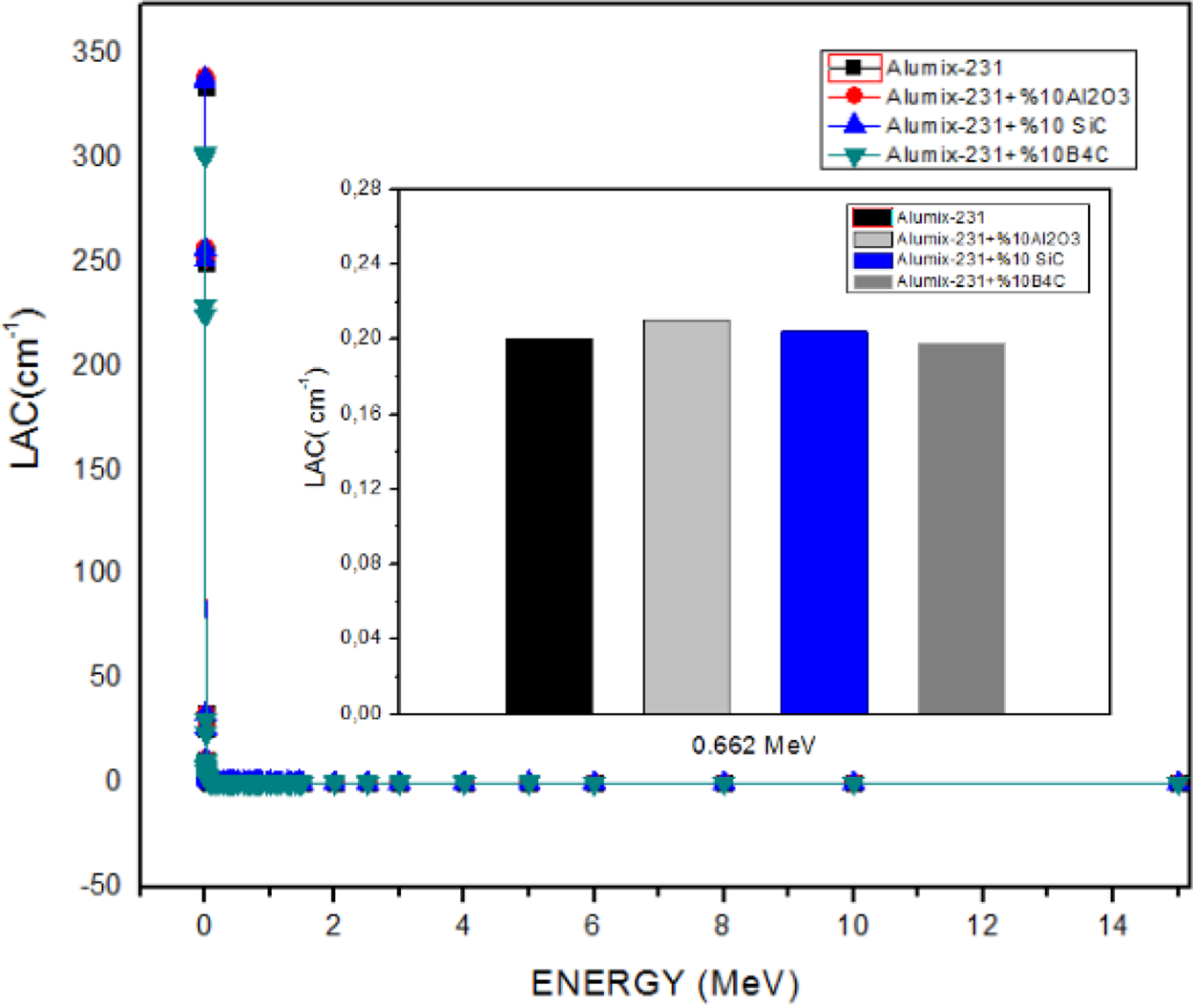
LAC values of Al_2_O_3_, SiC, and B_4_C (10%) reinforced Alumix-231 composite materials.

**Figure 14:**
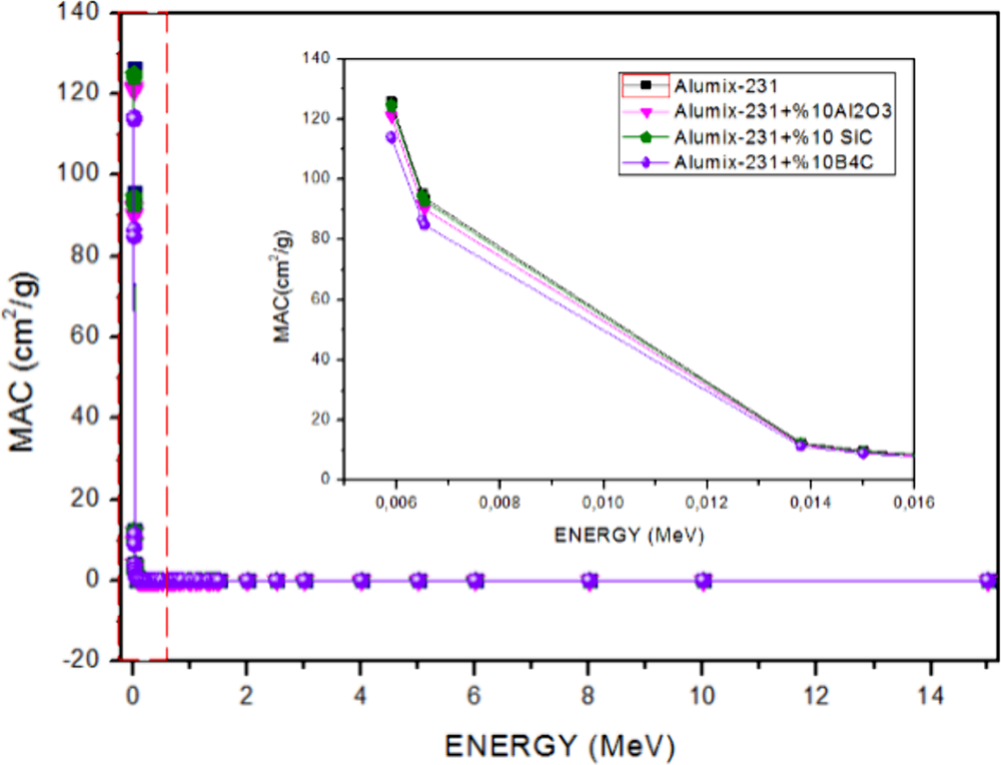
MAC values of Al_2_O_3_, SiC, and B_4_C (10%) reinforced Alumix-231 composite materials.

Theoretical density values of composite materials were used during the theoretical calculation. LAC and MAC graphs of the material are given in Figures 13 and 14, respectively. When examined from a theoretical point of view, the LAC value varies between 339.8 and 0.058 cm^–1^, and the MAC value varies between 126.217 and 0.022 cm^2^/g for all samples in the energy range of 5.89 × 10^–3^ to 15 MeV. Among the samples obtained by adding different ceramic materials into Alumix-231 without reinforcement material, the sample with the lowest LAC value, the Alumix-231 + 10% B_4_C composite material, is quite distinctly different from other samples. Although the LAC values of the other three samples are quite close to each other, we can compare them as 10% SiC + Alumix-231 < 10% Al_2_O_3_ + Alumix-231 < Alumix-231, in case a ranking is required. The main reason why the sample with the smallest value among the LAC values is Alumix-231 + 10% B_4_C is that the atomic number of B_4_C (B: 5, C: 6) is lower than other ceramic materials. The large atomic number of the material chosen as gamma shielding improves the permeability of the material against radiation. Depending on the increase in photon energy, the LAC values of the samples decrease. The photoelectric effect (<0.512 MeV) occurred at low energy levels, while pair production occurred (>1.02 MeV) in high energy regions. When the graph showing the MAC values obtained by dividing the LAC values by the densities of the samples (Figure 13) is examined, it is seen that the MAC value order is Alumix-231 + 10% B_4_C < Alumix-231 + 10% Al_2_O_3_ < Alumix-231 + 10% SiC < Alumix-231. Among the samples, the sample with the lowest MAC value was Alumix-231 + 10% B_4_C, while the sample with the highest value was the Alumix-231 material. Unlike MAC ordering, the reason why the SiC value is higher than Al_2_O_3_ in LAC ordering is due to the higher number of electrons per density. The reason why MAC values are very close to LAC values is due to the fact that the theoretical densities of the materials are very close.

**Figure 15:**
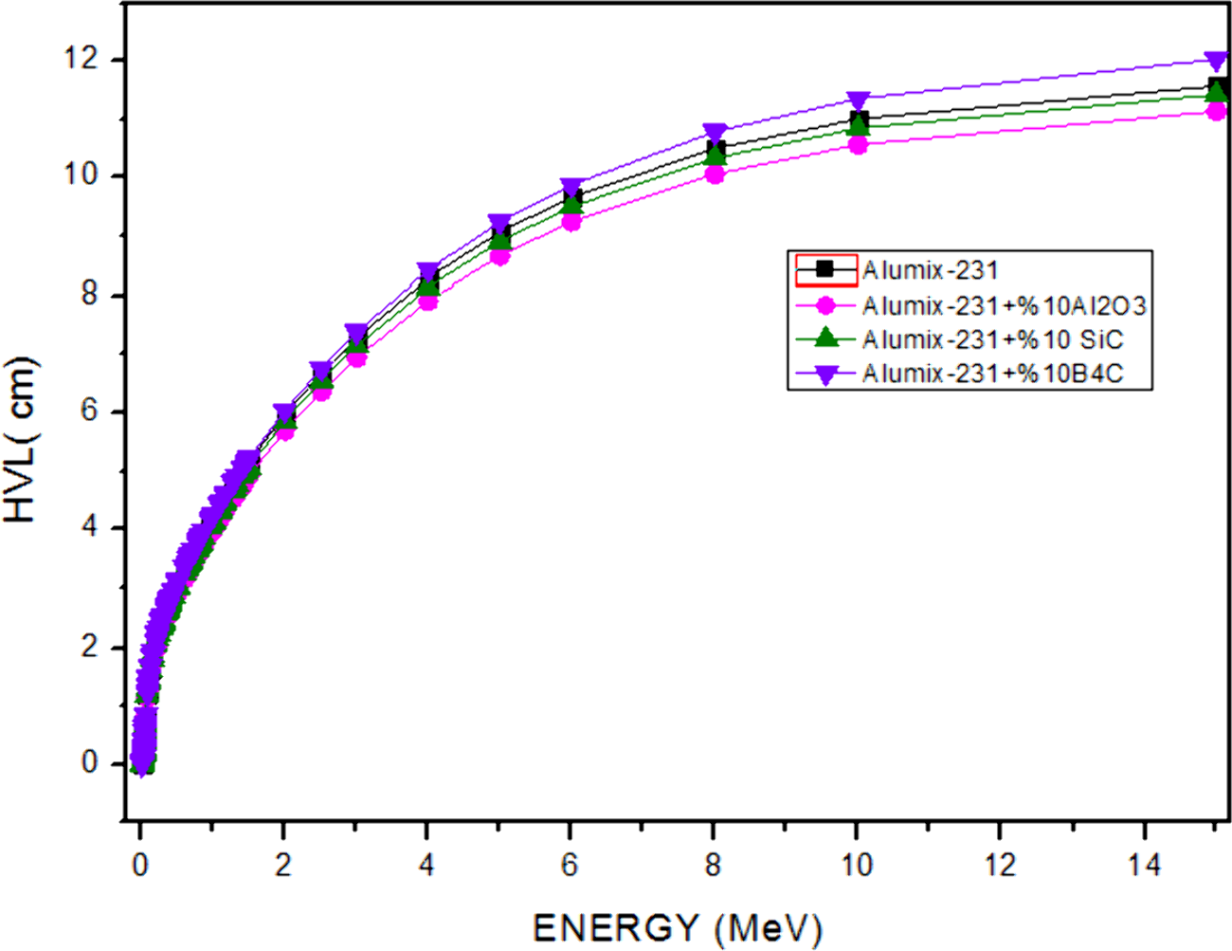
HVL values of Al_2_O_3_, SiC, and B_4_C (10%) reinforced Alumix-231 composite materials.

**Figure 16:**
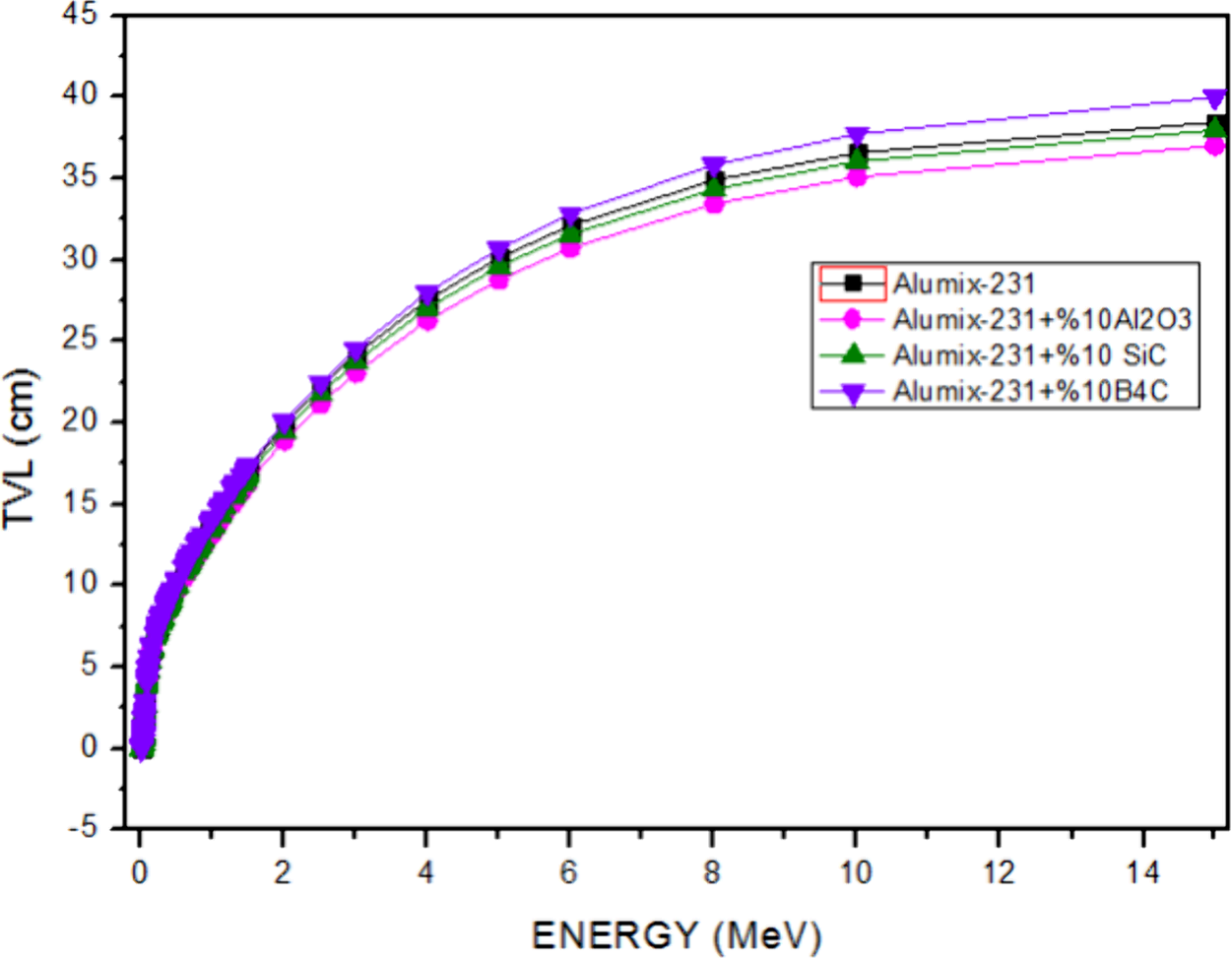
TVL values of Al_2_O_3_, SiC, and B_4_C (10%) reinforced Alumix-231 composite materials.

The material thickness values (HVL (Figure 15) and TVL (Figure 16)) required for radiation shielding showed a continuous increase, depending on the increase in energy. Especially in high energy regions where double formation (*E* > 1.02 MeV) is possible, it has taken very high values in required material thicknesses. When the HVL and TVL values of the materials were examined theoretically, they were calculated as 0.02–12.035 and 0.007–39.980 cm, respectively, in the energy range of 5.89 × 10^–3^ to 15 MeV. As can be seen in the HVL and TVL graphs, the required material thickness for material shielding is required the most for the Alumix-231 + 10% B_4_C composite sample. The main reason is that the atomic number and density value of B_4_C reinforcement material added to Alumix-231 are lower than those of reinforcement materials. In the Alumix-231 + 10% B_4_C composite sample, the HVL value was calculated as a maximum of approximately 12 cm, while the TVL value was calculated as a maximum of 40 cm. Alumix-231 + 10%Al_2_O_3_ is the material with the best shielding properties since it has the lowest HVL and TVL values. The maximum values of HVL and TVL of this sample are 11,138 and 37 cm, respectively.

**Figure 17:**
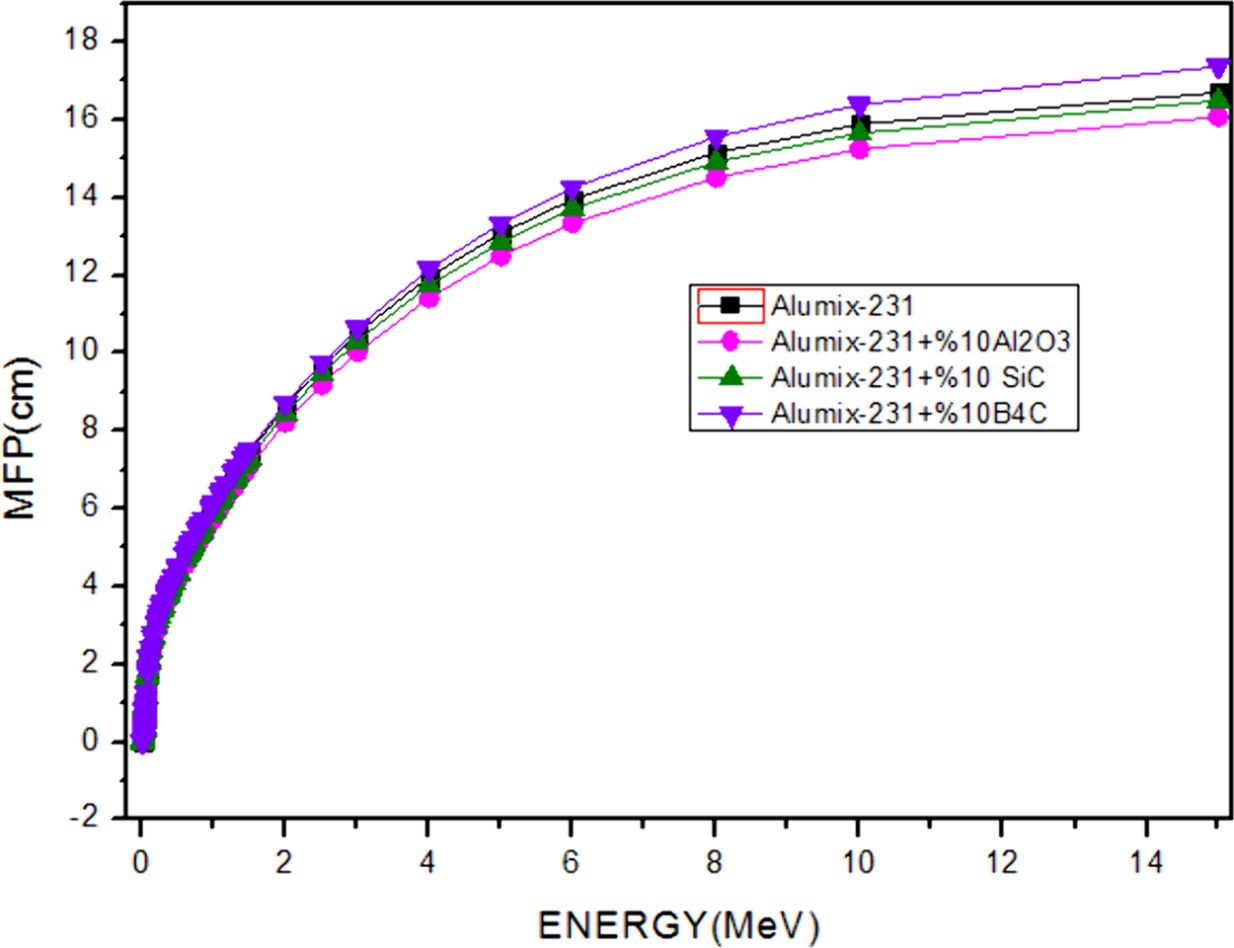
MFP values of Al_2_O_3_, SiC, and B_4_C (10%) reinforced Alumix-231 composite materials.

The MFP graph (Figure 17) parallels the HVL and TVL graphs. The MFP value increases with the increase of photon energy. In the energy regions where the photoelectric effect occurs (*E* < 0.512 MeV), which is the low energy region where the material is exposed to radiation, the distance required for two photon collisions to occur in succession is lower, while the required distance value increases at high energies where Compton scattering and pair production occur. While the maximum MFP value of the Alumix-231 + 10% B_4_C composite sample was 17,363 cm, the maximum value of the Alumix-231 + 10%Al_2_O_3_ MFP was calculated as 16,069 cm. According to these results, it has been shown that the Alumix-231 + 10%Al_2_O_3_ material has the best shielding properties since it has the lowest MFP value. The LAC value is inversely proportional to HVL, TVL, and MFP values. The higher the LAC value of a material, the lower the HVL, TVL, and MFP values. This shows that the material has a good shielding ability even at less thickness.

### 3.4 Experimental Radiation Transmittance Results

**Figure 18:**
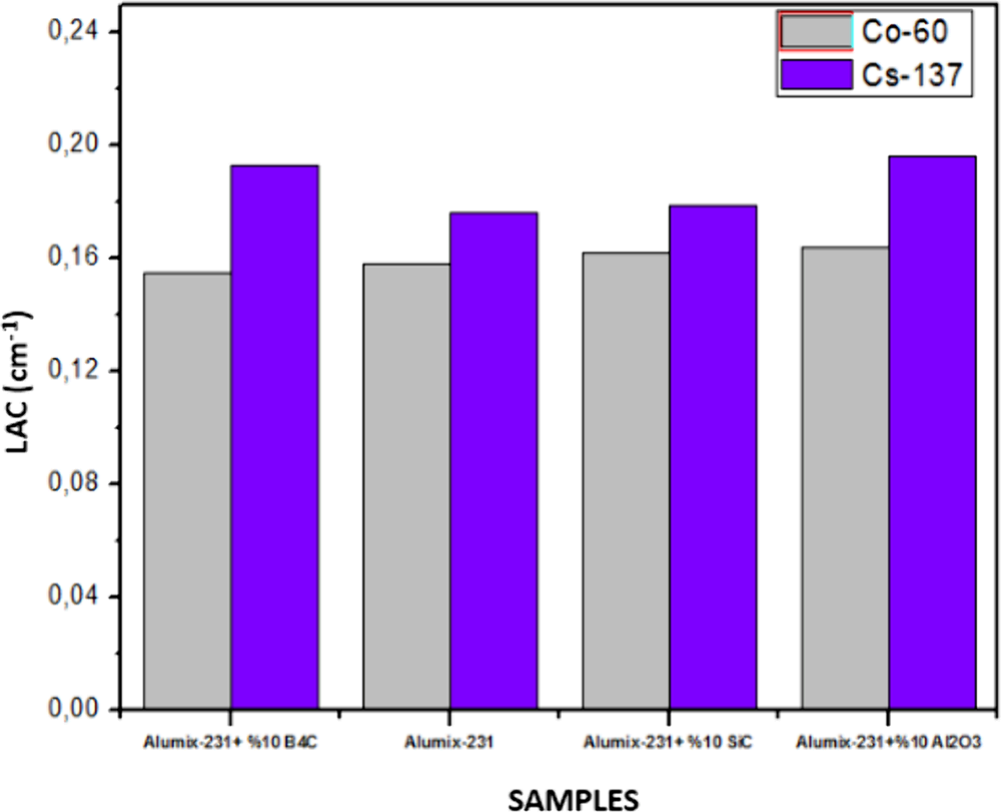
LAC values of Al_2_O_3_, SiC, and B_4_C (10%) reinforced Alumix-231 composite materials (experimental).

**Figure 19:**
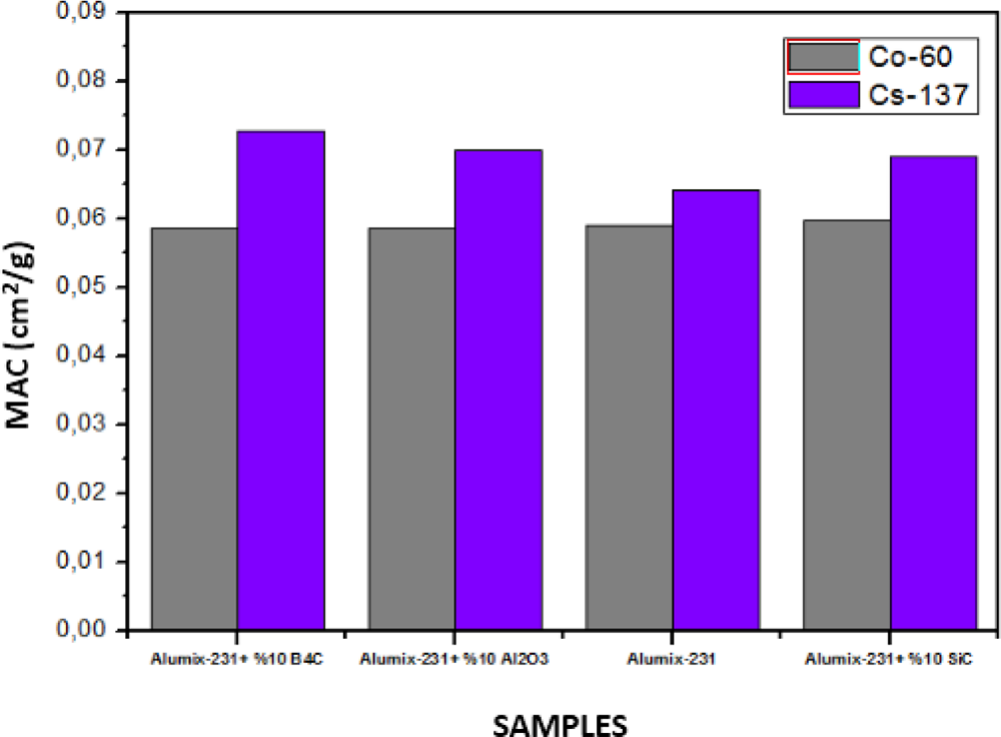
MAC values of Al_2_O_3_, SiC, and B_4_C (10%) reinforced Alumix-231 composite materials (experimental).

**Figure 20:**
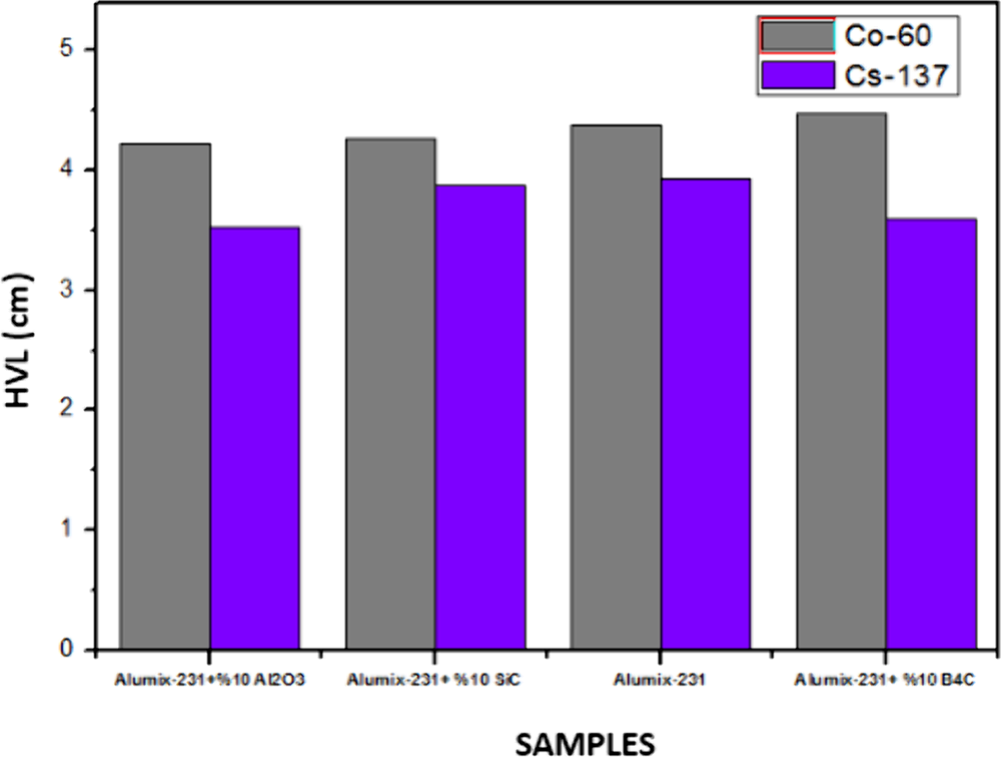
HVL values of Al_2_O_3_, SiC, and B_4_C (10%) reinforced Alumix-231 composite materials (experimental).

**Figure 21:**
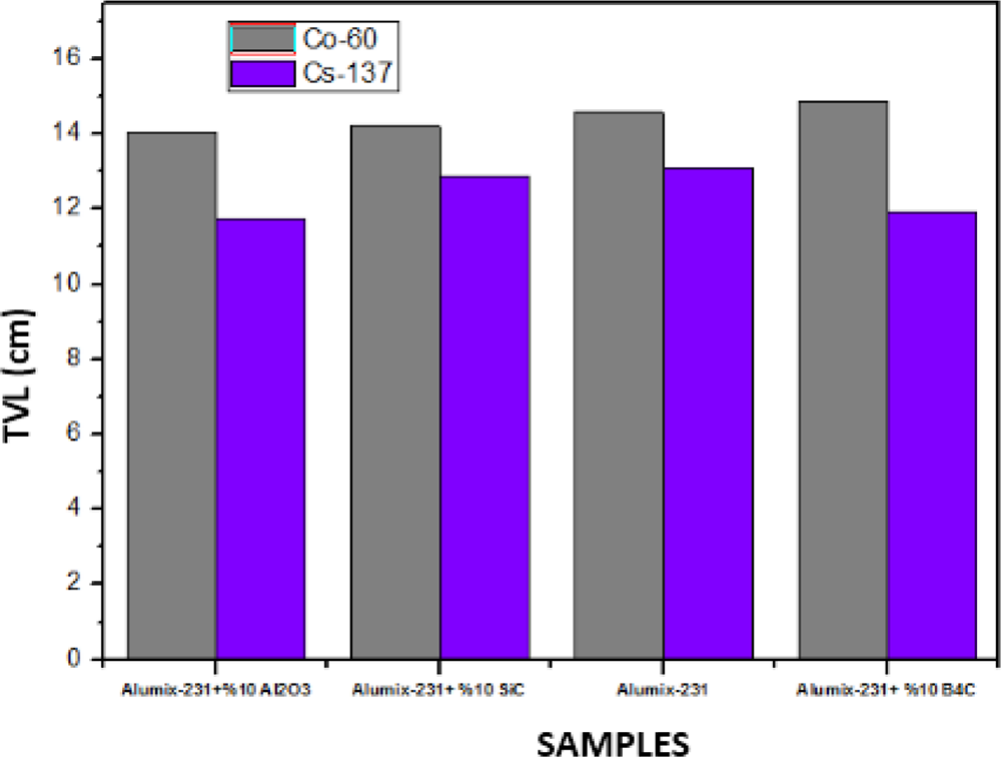
TVL values of Al_2_O_3_, SiC, and B_4_C (10%) reinforced Alumix-231 composite materials (experimental).

**Figure 22:**
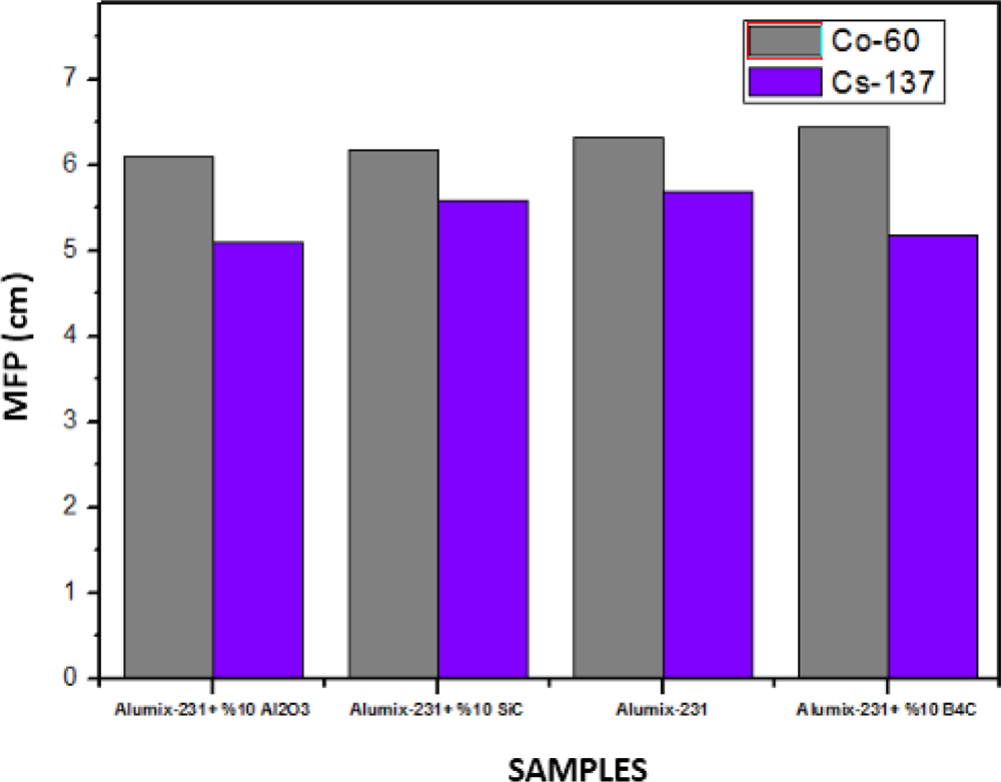
MFP values of Al_2_O_3_, SiC, and B_4_C (10%) reinforced Alumix-231 composite materials (experimental).

In the graphs given below, MAC, LAC, HVL, TVL, and MFP graphs are drawn by using eqs 1–5 of the data obtained as a result of radiation transmittance tests performed on Co-60 and Cs-137 γ radiation sources (Figures 18–22).

When the graphics are examined, it is seen that the samples show a better shielding performance against Cs-137. This is because Cs-137 γ radiation has a lower energy than Co-60. Materials have a weaker shielding ability at high energies. While the Alumix-231 + 10% Al_2_O_3_ material has the highest LAC value in Co-60 and CS-137 sources, Alumix-231 + 10% B_4_C has the lowest LAC value. It is due to the fact that the Al_2_O_3_ (Al: 13, O: 8) ceramic material consists of elements with the highest atomic numbers compared to other ceramic materials. The reason why the MAC values obtained as a result of the experimental studies are different from the values obtained by the theoretical calculations is that while the theoretical densities of the materials used during the production are quite close to each other, the experimental density values of the materials have changed due to the processes occurring during the production stages.

Although this did not create a big difference between the theoretical results of the samples and the experimental results, it caused the results to change. It is seen that the HVL and TVL values for Co-60 and Cs-137 gamma sources are Alumix-231 + 10% Al_2_O_3_ for the lowest material and Alumix-231 + 10% B_4_C for the highest values. According to these results, it is concluded that the Alumix-231 + 10% Al_2_O_3_ material is the best shielding material for Co-60 and Cs-137 gamma sources among the materials examined since it has the smallest HVL and TVL values. The Alumix-231 + 10% B_4_C material, on the other hand, has been shown to be the material with the lowest shielding properties since it has the highest HVL and TVL values. When the theoretical and experimental results are compared, it is seen that the results are concordant, but there are small differences. The reasons for the formation of these differences are thought to be the grain sizes of the powder materials, the discontinuities that occur during the production of the material blocks, and the presence of porosity. When the theoretical and experimental studies are examined, it is concluded that the Alumix-231 + 10% Al_2_O_3_ material is the best material to be used in gamma shielding.

## 4 Conclusions and Discussion

Within the scope of this study, composite samples were produced by adding 10% by weight Al_2_O_3_–SiC and B_4_C ceramic particles separately into Alumix-231 and Alumix-231 matrices, which do not contain any reinforcement material, using the powder metallurgy method. Microstructures (SEM and EDS) of these produced samples, theoretical radiation permeability properties using the Phy-X/PSD program, and experimental radiation permeability properties using Co-60 and Cs-137 radioactive sources were investigated. Among the samples produced, the material with the highest density was the Alumix-231-based 10% Al_2_O_3_ particle-reinforced composite sample. When the SEM images of the produced materials were examined, it was determined that the SiC ceramic phase was mostly in pointed, sharp-edged, or polygonal structures, B_4_C was in oval morphology, and the Al_2_O_3_ ceramic phase was in bright and irregular shape morphology.

Theoretical and experimental results are in good agreement with each other. LAC and MAC values of materials are higher at low radiation energies. Depending on the increase in the radiation energy, the LAC and MAC values of the materials decrease. According to the theoretical and experimental results, the Alumix-231 + 10% B_4_C composite material with the highest HVL and TVL values has the lowest gamma shielding ability, while the Alumix-231 + 10% Al_2_O_3_ material with the lowest HVL and TVL values has the highest gamma shielding ability. As a result, the materials have lower absorption coefficients but higher HVL and TVL values for the higher energy gamma radioisotope source.

The fact that the density values of the produced composite materials are high indicates that the mechanical properties of the materials can be prioritized. The investigation of the mechanical properties will better reveal whether there is a material that can replace concrete materials with weak mechanical properties, which are widely used today.

Thus, not only in nuclear technology but also in space technology, nanotechnology, etc., this is a study that can be used in advanced technologies as such.
